# Evidence in Asian Food Industry: Intellectual Capital, Corporate Financial Performance, and Corporate Social Responsibility

**DOI:** 10.3390/ijerph17020663

**Published:** 2020-01-20

**Authors:** Cheng-Hung Tsai, Eugene Burgos Mutuc

**Affiliations:** 1 Department of Business Administration, Cheng Shiu University, Kaohsiung 83347, Taiwan; 2 Postgraduate Program in Management, I-Shou University, Kaohsiung 840, Taiwan; eugene.mutuc@bulsu.edu.ph; 3 College of Business Administration, Bulacan State University, Malolos 3000, Philippines

**Keywords:** corporate social responsibility, intellectual capital, corporate financial performance, food industry, Asian context

## Abstract

Intellectual capital (IC) and corporate social responsibility (CSR) provide a strong link between the enterprise and stakeholders. These strategic approaches are responsible in value formation for better financial performance. This study investigates the mediating effects of corporate financial performance on the relationship between IC components (ICs) and CSR of firms from the food industry in Asia. We analyzed 308 firm-year observations of 44 listed firms from 2011 to 2017. The results of this study provided mixed findings regarding the effects of ICs and CSR. In addition, results vary from the disaggregated effects of each IC component on environmental, social, and governance pillars. The results also indicate that the combination of accounting and market-based estimates of financial performance was found to be significant mediating factor to explain the phenomenon which varies per ICs and dimensions of CSR. Lastly, the implications for sustainable business practices and investments in knowledge-based resources in the food industry are elaborated.

## 1. Introduction

Corporate social responsibility (CSR) has become a fast-growing essential requirement for a long-lasting enterprise in the recent decade. Consumers and governmental organizations have an increasing demand for CSR programs, nowadays. CSR is the allusion of firm behavior and one of the major issues in the business environment. It deals with a firm’s relationship toward stakeholders and the increasingly recognized moral implications in investments [1]. In addition, preservation of the environment, social participation, and good governance in business operations and reciprocity with their stakeholders are the basis of firms’ citizenship and voluntary initiatives [2,3]. The significant effect of CSR on financial performance has an overriding relevance on businesses, society, and nation-building [4].

Based on the landscape of literature on CSR, related issues and phenomenon have been discussed in general context. The investigation of this matter is relevant to the increasing recognition of CSR performance in a specific industry [5]. There are few studies which contemplate the analysis of CSR into sustainability and the cognizance of this strategy in a specific sector, with firms as the evaluating unit [6]. For instance, there is an increasing awareness from consumers and other stakeholders of firms from food industry regarding their purchasing power on reducing the demand for goods perceived as sustainable over non-sustainable [5]. In addition, Cuganesan et al. [5] mentioned that governments around the world are taking initiatives and actions in promoting CSR. This study extends the arguments of how CSR performance differs across industries to how it might differ within industries.

Firms from food industry have started to notice complexities in sustainability in terms of social, environmental, and economic aspects [6]. The perception and criticism of consumers regarding insufficient CSR programs can be destructive to a company [7]. In addition, the integration of supply chain accountability into CSR causes challenging issues in the management of socially responsible programs [7]. Rana et al. [6] mentioned that an unlimited case of issues such as labor practices and relationship between firms and community are accompanied by opportunities for a better standard of living through increased access to knowledge and technology. In addition, Rana et al. [6] alluded that the concern on community relations is escalated in the food industry of developing economies. The rising importance and relevance of such issues in the progression of the industry landscape, has become a key factor in business growth and strategy [6]. 

Value creation is the main concern in the effective utilization of the resources of a company, aligned from formulation of strategic programs and policies. Intellectual capital (IC) exists in all organizations as a stock of knowledge-based resources which an organization potentially can use in its value creation process [8]. Ethiraj, Kale, Krishnan, and Singh [9] and Haas and Hansen [10] mentioned that IC is an essential intangible requirement in the formation of corporate value. However, the increasing recognition of CSR programs with strong emphasis on social and environmental concerns to the conduct of business operations, for the purpose of solving issues in society [11], creates a huge debate and comprises the management of stakeholder relationships [12]. CSR can be associated as a strategic program of firms when it supports core business activities in promoting effectiveness and efficiency of firms to achieve its goal and generate substantial business-related benefits [13]. In fact, the relevance of CSR has attracted the attention of academics, practitioners, and policy makers [14]. 

Most of the prior studies reveal that CSR is a definitive factor of financial performance. Other facets of this phenomenon have been uncertain [15]. Moreover, prior research projects reveal that IC increases the value of the company and generates profitability [16,17]. The uncertainty in the past literature offers a great chance to examine the phenomenon with comprehensive reflections and estimates. We conjecture that the cognizance of the phenomenon is relevant and useful in promoting IC and CSR activities among business firms. Following the proposition of Razafindrambinina and Kariodimedjo [18], we contemplate that investigating corporate financial performance in an empirical study would deliver a better cognizance in a correlative study of CSR. Hence, we propose that a major issue in the pervasiveness of IC is its impact on CSR and this relationship is mediated by corporate financial performance.

To address these important issues, we developed an empirical study to fill the gaps in the literature. This study aims to investigate the phenomenon through the mediating role of corporate financial performance in the relationship between IC and CSR. CSR is a factor in improving IC which leads to better financial performance [4]. We conjecture that the effective and efficient utilization of IC would cause profitability advantage and CSR performance, eventually. We examine the phenomenon in the context of firms from the food industry in Asia. Food industry has substantial visibility to the public and plays a large role in daily human life and the economy. This industry also contends many CSR challenges such as food safety, obesity, abuse of alcohol, and packaging management [5]. The Center for Livable Future [19] mentioned that food sustainability-related issues are a concern for American voters. In the USA and Europe, food citizenship is a common concept [20]. Morin [20] added that expectations of the majority of Asian consumers are changing. Asia is mostly composed of developing countries. Recently, lawmakers, businesses, and mass media are attentive to the issues of poor labor practices and working conditions in developing economies [21]. For instance, the cost reduction of materials and processes by transferring the production to developing countries, increases the problem of food safety [22]. In addition, Perkowski [22] mentioned that the profitability of firms from developing countries is associated with the low cost of production but it affects the environment due to carbon footprint caused by logistic complexities.

Our main analysis focuses on the effects of IC components (ICs) such as human capital efficiency (HCE), structural capital efficiency (SCE), and capital employed efficiency (CEE) on the composite ratings of CSR. We also reflect on three pillars of CSR namely: environmental, social, and corporate governance to disaggregate the effects of the combined CSR scores. Moreover, this study contemplates the mediating effects of return on assets (ROA) and Tobin’s Q as proxies of corporate financial performance. This research article endows to the body of knowledge of firms’ efficiency and strategic approaches for the formation of value to firms from the food industry. We conduct an extensive knowledge generation in the literature on the relationships between IC and corporate social responsibility of firms from food industry, an industry currently facing sustainability challenges. Our evidence provides enlightenment to this industry about the beneficial roles of IC in implementing corporate social responsibility activities. Lastly, our empirical findings serve as a guide to the management of firms from food industry in utilizing resources through IC investments and promoting CSR programs which create value for the business and stakeholders. 

The remainder of the paper is organized as follows: Section 2 briefly discusses and reviews the empirical literature regarding IC, financial performance, and CSR. It also presents the formulated hypotheses of the study. Section 3 expounds the methodology applied in this study. Section 4 presents the empirical results and discussions. Lastly, Section 5 concludes the paper and recommends further studies.

## 2. Literature Review and Hypotheses Development

### 2.1. Intellectual Capital

Inkinen [23] and Wang, Wang, Cao, and Ye [24] discussed that IC is a multidimensional construct which varies from one idea to another. The emerging standard of approach in categorizing IC is a three-dimensional classification which includes human, structural, and relational capital [25,26,27,28,29,30]. These dimensions have strong relevance in the integration of knowledge to the employees of the company (their knowledge, skills, experience, expertise, innovation and learning capabilities, motivation); organizational structures, processes and information technology (software databases, process and project descriptions, intellectual properties, organizational culture, non-human storehouses of knowledge); and relationships and networks (connections with its customers, suppliers, partners, creditors, local community, and all internal connections within the company) [26,27,31,32,33]. 

### 2.2. Value-Added Intellectual Capital

Theorists and practitioners have made progress in proposing models to measure IC and its components [26,30,31,34,35]. The value-added intellectual coefficient (VAIC™) model is the most common accounting-based method to measure IC and its components, which was developed by Ante Pulic in 1993 [16]. Aras et al. [36] mentioned that this estimate is used frequently in finance literature. Pulic [35] used HCE and SCE as components of IC, and assessed capital employed (CE) as an additional efficiency. Abdulsalam, Al-Qaheri, and Al-Khayyat [37] mentioned that this is a suitable and impartial method with no subjective grading which is one of the advantages of this method.

### 2.3. Corporate Social Responsibility

Based on stakeholders’ perspective, CSR programs create a culture of citizenship to satisfy the stakeholders and generate favorable outcomes to the company [14,38]. Freeman [39] expounded that shareholders and other stakeholders, including the employees, consumers, vendors, and society shall be satisfied in the formulating and execution of company policies. Aguinis and Glavas [40] mentioned that CSR is an organizational activity which caters to the expectations of stakeholders regarding economic, social, and environmental engagements. Firms’ engagement in CSR activities promotes the relationship between firms and stakeholders [41]. Musibah and Alfattani [42] alluded that interests of stakeholders should be equally recognized aside from the maximization of the return to shareholders.

### 2.4. CSR Pillars

Based on various interpretations in the literature, CSR is a multi-dimensional construct. The expectations of stakeholders regarding economic, social, and environmental engagements are the main concerns of CSR [40]. Nowadays, firms are engaged in socially- and environmentally-responsible activities along with the other non-financial and financial dimensions to acquire the benefits of sustainable strategies [43]. Hence, environmental, social, and governance (ESG) reports are the means of firms to express their sense of responsibility to their stakeholders [44]. Kocmanová and Dočekalová [45] explained that the ESG key indicators present the measure in attaining sustainability. The key performance indicators (KPIs) for the three groups are as follows: (i) environment: global warming, environmental management systems and enforcement, efficiency, and other related issues; (ii) social status: employment health and safety, manpower, and stakeholder control; and (iii) governance: board effectiveness and corporate conduct. ESG are specific criteria to estimate the sustainability of a company. Iamandi, Constantin, Munteanu, and Cernat-Gruici [46] explained that these criteria are integrated into firms’ operation decision-making processes to conform to sustainable development goals and enhance their efficiency and image in the market.

### 2.5. Intellectual Capital and CSR

IC exists in all organizations as a stock of knowledge-based resources that an organization potentially can use in its value creation process [8]. It consists of several groups of interconnected knowledge-based resources, which make the basis of IC taxonomy. CSR, as a channel of valuable resources of a firm, generates positive outcomes such as better brand image and corporate reputation which improve competitive advantage and financial performance, subsequently [47,48,49,50]. These channels of maintaining and improving the reputation of a firm are in line with the resource-based view. Barney [51] explained that firms are dependent on different sets of resources and capabilities which include physical assets, raw materials, and intangible assets such as image, work environment, and human capital.

The IC of an organization is a relevant factor in its transition from the industrial age to the information age [12]. However, the increasing recognition of CSR programs with strong emphasis on social and environmental concerns to the conduct of business operations for the purpose of solving issues [11], creates a huge debate and comprises the management of stakeholder relationships [12]. Musibah and Alfattani [42] explained that CSR produces internal advantages related to the improvement of additional resource and opportunities and external advantages related to the relationship with stakeholders and the improvement of the reputation of the company. Intangible resources play an important role in improving firm’s CSR performance [38,52]. Musibah and Alfattani [42] mentioned that CSR contains a set of favorable implications in all three IC dimensions (human, organizational, and relational).

Frey et al. [53] investigated the relation of IC and CSR of Italian universities. Their study mentioned that there are overlapping areas in the IC and CSR human capital component and recognized that intangible assets are one of the leading indicators of value creation. Moreover, Razafindrambinina and Kariodimedjo [18] conducted an exploratory study which examines whether there is an association between IC and its components and corporate social responsibility disclosure of listed firms in Indonesia. Their study provided findings regarding the insignificant effect of the aggregate VAIC™ model and its components such as HCE and SCE on CSR disclosure. Their study concluded that the idea of CSR is implemented on an ad-hoc basis rather than treated as a business strategy. Furthermore, Musibah and Alfattani [42] determined the influence of IC on the CSR of Islamic Banks over the period of 2007–2011. Their study presented results that among the components of IC, CEE and SCE positively influenced CSR. In addition, their study found that financial performance has a significant mediating effect on intellectual components such as CEE and SCE, and CSR.

Prior literature provides evidence that IC has a positive effect on corporate financial performance [54,55]. In addition, early studies showed that CSR has a positive impact on corporate financial performance [1,18,47,48,49]. Hence, we hypothesize that IC has a positive relation on CSR, following the study of Razafindrambinina and Kariodimedjo [18] and Musibah and Alfattani [42]. We conjecture that the different factors of the management and maintenance of IC are interdependent towards CSR programs of firms from the food industry in Asia. We aim to identify the disaggregated effects of IC on CSR and its pillars, as CSR and IC as a whole, are similar in nature [51]. We conjecture that HCE, SCE, and CEE have favorable implications on CSR and its pillars such as environmental (ENV), social (SOC), and governance (GOV). Hence, we develop the following hypotheses:

**Hypotheses** **1 (H1a).** *There is a positive relationship between ICs and CSR.*

**Hypotheses** **1 (H1b).** *There is a positive relationship between ICs and ENV.*

**Hypotheses** **1 (H1c).** *There is a positive relationship between ICs and SOC.*

**Hypotheses** **1 (H1d).** *There is a positive relationship between ICs and GOV.*

### 2.6. Intellectual Capital and Financial Performance

According to the recent review of empirical research on IC and firm performance [23], the interactions between IC and performance outcomes have been increasingly studied since the early 2000s. IC is another driver for various organizational capabilities which bolster firm performance [56,57,58]. The present study reflects on the usability and efficiency of knowledge-based resources through the IC of companies to assess their influence on the relationship between CSR and corporate financial performance.

Previous empirical studies found that IC has an impact on firm performance based on the combinations and interactions of different ICs [59,60,61,62]. Hence, findings from prior studies show a positive and significant relationship between IC and corporate performance [54,55]. Chen, Cheng, and Hwang [17] provided evidence that a firm’s IC is positively associated with market value, financial performance, and is an indicator of future financial performance. Similarly, Tan et al. [54] conjectured that IC is positively related to future financial performance. Moreover, the aggregate IC efficiency is positive and significantly associated with market performance of multinational research and development (R&D) firms in the USA [63]. 

This study also conjectures that each component of IC has a positive link to corporate financial performance in terms of ROA and Tobin’s Q, following the notion of Musibah and Alfattani [42]. For instance, Goh [64] and Barathi Kamath [65] found that the productive utilization of tangible and intangible assets (HCE and CEE) has a great impact on corporate finance. In addition, Ahmed, Ahmed, Luqman, and Arshad [66] mentioned that ICs’ performance is very important for the survival and reliability of a company based on the strongly perceived impact of IC and its components (HCE, SCE, CEE, and VAIC™) on financial performance. Furthermore, resource-based theory explained that the appropriate use of structural capital generates greater financial performance [42]. We conjecture that HCE, SCE, and CEE have favorable impacts on financial performance in terms of ROA and Tobin’s Q. Therefore, this study develops the following hypothesis:

**Hypothesis 2** **(H2).** *There is a positive relationship between IC and financial performance*.

### 2.7. Financial Performance and CSR

Early studies have shown that CSR has a positive impact on corporate financial performance [1,47,48,49,67]. Kim, Park, and Wier [68] considered financial performance as a variable that might affect the relationship between CSR and earnings management. They conjectured that firms with better financial performance are capable of implementing CSR programs and activities. Similarly, we hypothesize that firm financial performance has a positive effect on CSR and its pillars. We postulate that CSR is responsible in the value creation of a company. Hence, we develop the following hypotheses:

**Hypotheses** **3 (H3a).** *The higher the financial performance, the higher the CSR ratings will be.*

**Hypotheses** **3 (H3b).** *The higher the financial performance, the higher the ENV ratings will be.*

**Hypotheses** **3 (H3c).** *The higher the financial performance, the higher the SOC ratings will be.*

**Hypotheses** **3 (H3d).** *The higher the financial performance, the higher the GOV ratings will be.*

### 2.8. IC, Financial Performance, and CSR

The impact of IC on CSR performance of firms is a remarkable concern among scholars according to the literature on business ethics and sustainability. Previous studies explained the phenomenon and the conduciveness of IC as a determinant of CSR. However, inconclusive results continue over the analysis of IC and CSR. Lin et al. [4], Jain et al. [69], and Khurshid et al. [70] discussed the interlinkages of IC, financial performance, and CSR and reflected IC as a mediator. Moreover, Pedrini [71] inferred that the ideal execution of socially responsible activities in improving knowledge-based resources results in favorable financial performance.

IC has an indirect impact on firms’ financial performance dependent on the business strategies utilized by the company [72]. However, no current research has been undertaken to investigate the mediating effect of financial performance on the IC and CSR relationship, which is the focus of this study. We believe that by using financial performance as a mediator of the IC–CSR relationship, we can provide a complete and better assessment of their correlations and deepen the assimilation of implications of knowledge-based resources on CSR. We consider financial performance as an essential factor to explain IC as an investment of the company to knowledge-based resources which leads to a better CSR rating. We argue that firms with better financial performance arbitrate the impact of the efforts of their company to have competent and motivated employees, high-quality knowledge management processes, and greater stakeholder-orientation on CSR strategies. The value of a company is an outcome of the firm’s investment in IC [64]. Bontis [73] mentioned that it is an essential element to achieve a competitive advantage compared to firms’ other resources. In this study, we conjecture that better ICs (HCE, CEE, and SCE) would result in better financial performance and a higher CSR rating, subsequently. Hence, we develop the following hypotheses:

**Hypotheses** **4 (H4a).** *Financial performance mediates the relationship between ICs and CSR.*

**Hypotheses** **4 (H4b).** *Financial performance mediates the relationship between ICs and ENV*.

**Hypotheses** **4 (H4c).** *Financial performance mediates the relationship between ICs and SOC.*

**Hypotheses** **4 (H4d).** *Financial performance mediates the relationship between ICs and GOV.*

**Figure 1:**
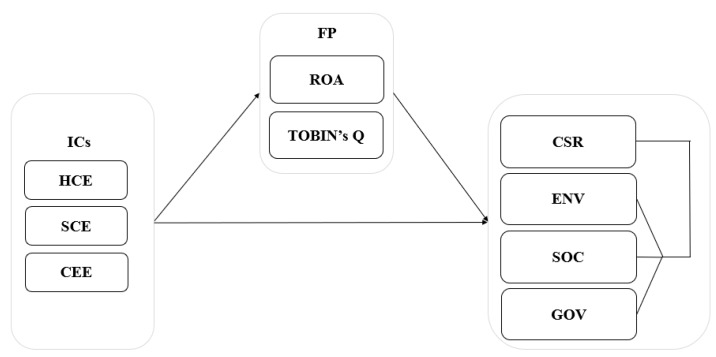
Research framework. Abbreviations: ICs: intellectual capital components; HCE: human capital efficiency; SCE: structural capital efficiency; CEE: capital employed efficiency; FP: financial performance; ROA: return on assets; CSR: corporate social responsibility; ENV: environmental; SOC: social; GOV: governance.

Figure 1 presents the framework of the study based on the hypothesized relationships. This framework presents the independent variables such as IC components namely: human capital efficiency (HCE), structural capital efficiency (SCE), and capital employed efficiency (CEE). The financial performance is the mediator, measured through accounting and combination of accounting (ROA) and market-based estimates (Tobin’s Q), and CSR and its dimensions namely: environment (ENV), social (SOC), and governance (GOV) are the dependent variables.

## 3. Research Methodology

### 3.1. Data and Sample

This study examines the mediating role of financial performance in the relationship between ICs and CSR of firms from food industry in Asia. These firms are listed in the Thomson Reuters ESG database. This study reflects on a final sample of 44 firms with 308 firm-year observations from 2011 to 2017. The CSR data of these firms were matched to other financial data from Thomson Reuters Eikon database. The observations with negative value-added (VA) scores were excluded due to the inability of the VAIC™ model to deal with the negative VA values, because “this would then mean that the company is expending more input resources than its output” [54,55].

### 3.2. CSR Ratings

This study contemplates on the ESG (environment, social, and governance) composite ratings as a proxy of CSR. These data are collected from S-Network File Transfer Protocol (FTP), an online database of ESG data from Thomson Reuters. The ESG composite rating is the combination of the average ratings of the three pillars and ESG controversies. These controversies are composed of disputes across the ten categories from environment, social, and governance pillars. Categories with higher weights are those items which contain different issues. For instance, the management category under the corporate governance pillar consists of multiple issues such as composition, diversity, independence, committees, and compensation. Thomson Reuters [74] explained that the environmental pillar is composed of resource use, emission reduction, and innovation categories while the social pillar is composed of workforce, human rights, community, and product responsibility categories. Moreover, the governance pillar consists of categories such as management, shareholders, and CSR strategies.

Thomson Reuters [74] defined the ten categories used in the measurement of each pillar. Under the environmental pillar, the resource use rating is composed of capability and performance of business firms to conserve resources and to improve supply chain management in an eco-efficient way, while the emission reduction rating is composed of commitment and firms’ effectivity to lessen environmental emission on production and operational processes. In addition, the innovation rating is composed of the capability of firms to lessen the costs related to environmental activities and new market opportunity creations.

Moreover, Thomson Reuters [74] explained that different considerations were considered in the social pillar measurement. Workforce rating measures the effectivity of a firm in providing a healthy and safe workplace, maintaining diversity and equal opportunities, and learning and development opportunities for its employees towards job satisfaction. The human rights rating measures the effectivity of the firm towards basic human rights consideration. In addition, the community rating covers the commitment of the company towards citizenship, public health protection, and ethics consideration. Lastly, the product responsibility rating measures the capability of a company to provide quality goods and services by incorporating health and safety, integrity, and data privacy of consumers.

Furthermore, Thomson Reuters [74] explained that different considerations were considered in the governance pillar measurement. Management rating covers the effectivity and commitment of a company in implementing corporate governance best practices while shareholders rating reflects the effectivity of a company to a fair treatment of shareholders and the use of anti-takeover devices. In addition, the CSR strategy rating covers the discussion of business firms regarding CSR implementation and its integration to economic, social, and environmental aspects into its day-to-day decision-making processes.

### 3.3. Corporate Financial Performance Measures

There is lack of consensus regarding the evaluation and measurement of a firm’s financial performance. However, different measures were previously employed such as accounting, market-based, and a combination of accounting and market-based measures [75]. The accounting measure captures the historical aspects of a firm’s financial performance such as return on equity (ROE), return on assets (ROA), return on sales (ROS), return on capital employed (ROCE), and earnings per share (EPS). However, market-based measures focus on a firm’s future performance as opposed to past performance such as investor returns. The combination of both accounting and market-based measures includes Tobin’s Q and stock returns which represent different perspectives on financial performance and have different implications [76]. Accounting-based measures provide information regarding the internal decision-making process of firms and the performance of its managers [77]. The present study utilizes the ROA measure, which represents the ratio between profits before tax to total assets. It also reflects the efficiency of a company to manage its assets to generate earnings. Moore [78] mentioned that the accounting-based measure is more appropriate in the analysis of the relationship between CSR and financial performance in terms of detection purpose. Moreover, this study utilizes Tobin’s Q, which represents the ratio between the market value of a firm’s physical assets and its replacement value [79]. The market value of a company’s assets is measured by the market value of its outstanding stock and debt, whilst the replacement cost of assets is measured using their book value. A ratio of 1 or more indicates that the firm’s market value exceeds that of its recorded assets. 

### 3.4. Intellectual Capital Measure

This study measures IC based on value-added intellectual capital (VAIC™) model. This model is widely adapted by numerous researchers and practitioners as a measure of knowledge-based resources of the company as IC is a multidimensional construct [4,16,54,80,81,82,83]. We draw on the subsequent steps in measuring VAIC™ following the approach of Firer and Williams [62], Pulic [16], Chen et al. [17], Nazari and Herremans [84], Zeghal and Maaloul [82], and Maditinos et al. [59], and Lin et al. [4].

First, we computed value added (*VA*) as the sum of interest expenses (*INT*), depreciation expenses (*DEP*), dividends (*DIV*), corporate taxes (*CT*), equity of minority shareholders in net income of subsidiaries (*MIN*), and profits retained for the year (*RE*) [4,63]:

*VA* = *INT* + *DEP* + *DIV* + *CT* + *MIN* + *RE* (1)

Second, we computed human capital efficiency (*HCE*) as the coefficient of the computed value added over human capital (*HC*). *HC* is embodied in employees and includes their expertise, experience, skills, and motivation [34]. Employee costs are used as a proxy of *HC* [64], where *HC* is measured through salaries and benefits of employees. We measure *HCE* as follows:

*HCE* = *VA/HC* (2)

Third, we computed structural capital efficiency (*SCE*) as the relationship between VA and structural capital (*SC*). *SCE* is computed as the ratio of *SC* to *VA*.

*SCE* = *SC/VA* (3)

Equation (3) shows that *VA* is the denominator and *SC* is the numerator which provides different implications from Equation (2). Pulic [16], Zeghal and Maaloul [82], and Lin et al. [4] mentioned that IC is mainly composed of human and structural capital. Lin et al. [4] noted that *HC* and *SC* are negatively correlated in creating value for firms. We calculated *SC* as follows:

*SC* = *VA/HC* (4)

Pulic [16] mentioned that a firm’s value creation does not just originate from a firm’s IC, but concluded that a company’s value creation principally originates from IC and physical capital.

Fourth, we calculated the contribution of physical capital (*CA*) utilized in a firm’s value creation. Capital employed efficiency (*CEE*) provides information regarding the ratio of value added over the employed invested capital. *CA* is measured by the book value of net assets. We calculated CEE as follows:

*CEE* = *VA/CA* (5)

The value of VAIC™ can be divided into three dimensions, namely: *HCE*, *SCE*, and *CEE*. These components represent the value created from the total resources of the company. 

### 3.5. Regression Models

This study employs a multivariate regression model to test the relationship between intellectual capital components and *CSR* and its pillars, and the mediating effect of corporate financial performance on the relationship between ICs and CSR and its pillars. We control several variables which are known to influence and provide other plausible explanations to induce the net effects of IC on CSR. We include firm size, leverage, and R&D intensity as control variables. We utilize the natural logarithm of total assets as a proxy of firm size [42,85,86]. In addition, we utilize leverage which represents the ratio of total liabilities to total assets [42,85]. Moreover, R&D intensity is the ratio of R&D expenses to total assets which measures firm’s activities in R&D from its resources and enhances productivity and generates firm value [4,87,88]. Lastly, this study includes institutional variables such as year, country, and industry to control for fixed effects in the regression analysis.

This study considers Baron and Kenny’s [89] proposed method to test the mediation hypothesis of financial performance (ROA and Tobin’s Q) on the relationship between ICs and CSR (and its pillars). In addition, we use the following conditions to establish mediation: (1) ICs must affect CSR and CSR pillars; (2) ICs must affect financial performance (ROA and Tobin’s Q); and (3) when CSR and the CSR pillars are regressed on each IC component and each financial performance proxy.

Hence, we estimate the following models:

(6) $$CSR_{i,t}=\;\alpha \;+\;\beta_{1}\;HCE_{i,t}\;+\;\beta_{2}\;SCE_{i,t}\;+\;\beta_{3}\;CEE_{i,t}\;+\;\beta_{4}\;LEV_{i,t}\;+\beta_{5}\;SIZE_{i,t}\;+\beta_{6}\;RDI_{i,t}\;+\;\sum^{\text{​}}YEAR_{i,t}\;+\sum^{\text{​}}COUNTRY_{i,t}\;+\;\varepsilon_{i,t}$$

(7) $$CFP_{i,t}\;=\;\alpha^{1}\;+\;\beta_{1}^{1}HCE_{i,t}+\;\beta_{2}^{1}\;SCE_{i,t}\;+\;\beta_{3}^{1}\;CEE_{i,t}+\beta_{4}^{1}\;LEV_{i,t}+\beta_{5}^{1}\;SIZE_{i,t}+\beta_{6}^{1}\;RDI_{i,t}+\;\sum^{\text{​}}YEAR_{i,t}\;+\sum^{\text{​}}COUNTRY_{i,t}\;+\;\varepsilon_{i,t}^{1}\;$$

(8) $$CSR_{i,t}\;=\;\alpha^{2}\;+\;\beta_{1}^{2}CFP_{i,t}+\;\beta_{2}^{2}\;LEV_{i,t}+\beta_{3}^{2}\;SIZE_{i,t}+\beta_{4}^{2}\;RDI_{i,t}+\;\sum^{\text{​}}YEAR_{i,t}\;+\sum^{\text{​}}COUNTRY_{i,t}\;+\;\varepsilon_{i,t}^{2}$$

(9) $$CSR_{i,t}\;=\;\alpha^{3}\;+\;\beta_{1}^{3}HCE_{i,t}+\;\beta_{2}^{3}\;SCE_{i,t}\;+\;\beta_{3}^{3}\;CEE_{i,t}+\beta_{4}^{3}\;CFP_{i,t}+\beta_{5}^{2}\;LEV_{i,t}+\beta_{6}^{2}\;SIZE_{i,t}+\beta_{7}\;RDI_{i,t}+\;\sum^{\text{​}}YEAR_{i,t}+\;\sum^{\text{​}}COUNTRY_{i,t}\;+\;\varepsilon_{i,t}^{3}$$

where *CSR* is the corporate social responsibility ratings based on environmental, social, and governance pillars ratings on return on assets as a proxy of corporate financial performance; *CFP* is the corporate financial performance (ROA and Tobin’s Q); ROA is the ratio between profits before tax to total assets; Tobin’s Q is a ratio between the market value of a firm’s physical assets and its replacement value; *LEV* is the ratio of total liabilities to total assets; *SIZE* is the natural logarithm of the total assets; *RDI* is the R&D intensity based on the ratio of R&D expenditure to total assets; and *ɛ_i_* is the residual in the regression. We conducted individual regression analyses for each pillar of CSR such as environmental (ENV), social (SOC), and governance (GOV) to disaggregate the effects of IC on CSR. *HCE* is the human capital efficiency; *SCE* is the structural capital efficiency; and *CEE* is the capital employed efficiency.

## 4. Results and Discussions

Table 1 presents the descriptive statistics and the correlation coefficients between all of the variables utilized in this study. Table 1 shows that CSR has a mean value of 47.62 based on the Thomson Reuters ESG database. The components of CSR show that SOC has the highest mean value of 56.04 while GOV has the lowest mean value of 33.40. Return on assets (ROA) shows a mean value of 6.35, which indicates that most of the firms from the food industry in Asia have an efficient utilization of assets to generate earnings. Tobin’s Q shows a mean value of 1.52 × 10^−3^, indicating that capital of firms from food industry is valued by the stock market less than its replacement cost. ICs such as HCE show a highest mean value of 11.23. CSR is significantly and positively related to its components. 

We conducted a separate regression for each component to identify the individual effect of ICs on each CSR pillar. CEE is significantly and positively related to CSR, implying that as the level of CEE increases, the value of CSR also increases. In addition, RDI, ROA, and Tobin’s Q are positively and significantly related to CSR. SCE has a positive and significant relation on ENV and SOC while CEE has a positive and significant relation on ENV and SOC. However, HCE is significantly and positively related to GOV, implying that as the human capital efficiency of firms increases, its corporate governance improves. 

We performed regression analysis to examine the mediating effect of corporate financial performance on the relationship between ICs and CSR and its pillars. Table 2 shows the regression results of the mediating effect of corporate financial performance on the relationship between ICs and CSR. Model 1 of Table 2 shows that CEE has a positive and significant effect on CSR with β = 0.19 at *p* < 0.01. This finding supports H1a which states that ICs (CEE) have a positive relationship with CSR. Our findings about the effect of CEE on CSR of firms from the food industry are similar to the result of firms from banking industry from the study of Musibah and Alfattani [26]. 

Model 2 presents the relationship between the components of IC and financial performance (ROA and Tobin’s Q). Table 2 shows CEE has a positive and significant effect on corporate financial performance represented by ROA and Tobin’s Q with β = 0.44 at *p* < 0.01 and β = 0.29 at *p* < 0.01, respectively. In addition, SCE has a positive and significant effect on Tobin’s Q with β = 0.41 at *p* < 0.01. These findings are consistent with H2 which states that there is a positive relationship between ICs and financial performance (ROA and Tobin’s Q). However, HCE has a negative and significant relationship with ROA and Tobin’s Q at β = −0.30 at *p* < 0.05 and β = 0.46 at *p* < 0.01, respectively. These findings are inconsistent with H2. Model 3 presents the relationship between the firms’ financial performance (ROA and Tobin’s Q) and CSR. Table 2 shows that Tobin’s Q has a negative and significant effect on CSR with β = −0.18 at *p* < 0.01. In addition, ROA has insignificant impact on CSR. These outcomes do not support H3a which states that the higher the financial performance, the higher the CSR will be. 

Model 4 presents the results of the mediating effect of corporate financial performance on the relationship between ICs and CSR. Model 4 of Table 2 shows that CEE has a positive and significant effect on CSR with β = 0.21 at *p* < 0.01. ROA shows an insignificant effect on CSR. Hence, ROA has no mediating effect on the relationship between ICs and CSR, inconsistent with H4a. Moreover, Model 4 of Table 2 shows that CEE has a positive and significant effect on CSR with β = 0.28 at *p* < 0.01. Tobin’s Q has a negative and significant effect on CSR with β = −0.29 at *p* < 0.01. Hence, Tobin’s Q partially mediates the relationship between ICs and CSR, consistent with H4a_._ This result provides evidence that financial performance in terms of the combination of accounting and market-based measures has an arbitrary impact on the relationship between the components of IC and CSR, a result conforming to the findings of Musibah and Alfattani [26]. However, HCE and SCE have an insignificant effect on CSR. Hence, Tobin’s Q has no mediating effect on the relationship between these ICs and CSR.

Table 3 shows the regression results of the mediating effect of corporate financial performance on the relationship between ICs and the environmental pillar of CSR. Model 1 of Table 3 shows that CEE has a positive and significant effect on ENV with β = 0.13 at *p* < 0.10. This finding supports H1b which states that ICs (CEE) have a positive relationship with ENV. Our findings about the effect of CEE on ENV of firms from the food industry indicate that a higher capital-employed efficiency of firms from the food industry is an advantage to conduct socially responsible activities for the environment. In addition, we postulate that the other intellectual components are not relevant to environmental CSR but have significance on the other CSR pillars.

Model 2 presents the relationship between the components of IC and financial performance (ROA and Tobin’s Q). Table 3 shows CEE has a positive and significant effect on corporate financial performance represented by ROA and Tobin’s Q with β = 0.44 at *p* < 0.01 and β = 0.29 at *p* < 0.01, respectively. In addition, SCE has a positive and significant effect on Tobin’s Q with β = 0.41 at *p* < 0.01. These findings are consistent with H2 which states that there is a positive relationship between ICs and financial performance (ROA and Tobin’s Q). However, HCE has a negative and significant relationship with ROA and Tobin’s Q at β = −0.30 at *p* < 0.05 and β = 0.46 at *p* < 0.01, respectively. These findings are inconsistent with H2. Model 3 presents the relationship between the firms’ financial performance (ROA and Tobin’s Q) and ENV. Table 3 shows that Tobin’s Q has a negative and significant effect on ENV with β = −0.14 at *p* < 0.10. In addition, ROA has an insignificant impact on ENV. These outcomes do not support H3b which states that the higher the financial performance, the higher ENV will be. 

Model 4 presents the results of the mediating effect of corporate financial performance on the relationship between ICs and ENV. Model 4 of Table 3 shows that CEE has a positive and significant effect on ENV with β = 0.15 at *p* < 0.10. ROA shows an insignificant effect on ENV. Hence, ROA has no mediating effect on the relationship between ICs and ENV, inconsistent with H4b. Moreover, Model 4 of Table 3 shows that CEE has a positive and significant effect on ENV with β = 0.20 at *p* < 0.01. Tobin’s Q has a negative and significant effect on ENV with β = −0.22 at *p* < 0.01. Hence, Tobin’s Q partially mediates the relationship between ICs and ENV, consistent with H4b_._ This result provides evidence that financial performance in terms of the combination of accounting and market-based measures has an arbitrary impact on the relationship between the components of IC and ENV. However, HCE and SCE have an insignificant effect on ENV. Hence, Tobin’s Q has no mediating effect on the relationship between these ICs and ENV.

Table 4 shows the regression results of the mediating effect of corporate financial performance on the relationship between ICs and the social pillar of CSR. Model 1 of Table 4 shows that CEE has a positive and significant effect on SOC with β = 0.25 at *p* < 0.01. This evidence supports H1c which states that there is a positive relationship ICs and SOC. HCE has negative and significant effect on SOC with β = −0.36 at *p* < 0.05, inconsistent with H1c. However, SCE has an insignificant effect on SOC. We conjecture that a higher capital-employed efficiency is an advantage to conduct socially responsible activities in firms from the food industry for the community, employees, and other related social aspects. In addition, we infer that the SCE is not relevant while HCE has an inverse impact on the social pillar of CSR. The social pillar is composed of workforce, human rights, community, and product responsibility categories. Human capital is embodied in employees and includes their expertise, experience, skills, and motivation [34]. The structural capital component of IC is focused on building infrastructure needed by human capital to create value. Hence, there is a trade-off between HCE, SCE, and SOC.

Model 2 presents the relationship between the components of IC and financial performance (ROA and Tobin’s Q). Table 4 shows CEE has a positive and significant effect on corporate financial performance represented by ROA and Tobin’s Q with β = 0.44 at *p* < 0.01 and β = 0.29 at *p* < 0.01, respectively. In addition, SCE has a positive and significant effect on Tobin’s Q with β = 0.41 at *p* < 0.01. These findings are consistent with H2 which states that there is a positive relationship between ICs and financial performance (ROA and Tobin’s Q). However, HCE has negative and significant relationship with ROA and Tobin’s Q at β = −0.30 at *p* < 0.05 and β = 0.46 at *p* < 0.01, respectively. These findings are inconsistent with H2. Model 3 presents the relationship between firms’ financial performance (ROA and Tobin’s Q) and ENV. This outcome does not support H3c which states that the higher the financial performance, the higher the SOC will be. Table 4 shows that ROA has a positive and significant effect on SOC with β = 0.14 at *p* < 0.10. In addition, Tobin’s Q has an insignificant impact on SOC.

Model 4 presents the results of the mediating effect of corporate financial performance on the relationship between ICs and SOC. Model 4 of Table 4 shows that CEE has a positive and significant effect on SOC with β = 0.24 at *p* < 0.01. HCE has a negative and significant effect on SOC with β = −0.35 at *p* < 0.10. However, SCE has an insignificant effect on SOC. ROA has an insignificant effect on SOC. Hence, ROA has no mediating effect on the relationship between ICs and SOC, inconsistent with our hypothesis. This finding provides evidence that financial performance in terms of the accounting measure has no arbitrary impact on the relationship between the ICs and SOC activities of firms from the food industry. Moreover, Model 4 of Table 4 shows that SCE and CEE have positive and significant effects on SOC with β = 0.41 at *p* < 0.05 and β = 0.31 at *p* < 0.01, respectively. HCE has a negative and significant effect on SOC with β = −0.45 at *p* < 0.05. Tobin’s Q shows a negative and significant effect on SOC with β = −0.20 at *p* < 0.05. Hence, Tobin’s Q has a partially mediating effect on the relationship between ICs and the social pillar of CSR, consistent with the hypothesis of the study. These findings provide evidence that financial performance in terms of the combination of accounting and market-based measure has an arbitrary impact on the relationship between ICs and SOC. 

Table 5 shows the regression results of the mediating effect of corporate financial performance on the relationship between ICs and the governance pillar of CSR. Model 1 of Table 5 shows that HCE, SCE, and CEE have an insignificant effect on GOV. These findings are not parallel to H1d which states that a positive relationship between ICs and GOV exists on firms from the food industry in Asia. We infer that the ICs are not relevant to the governance pillar of CSR. The governance pillar is consisted of categories such as management, shareholders, and CSR strategies. 

Model 2 presents the relationship between the components of IC and financial performance (ROA and Tobin’s Q). Table 5 shows CEE has a positive and significant effect on corporate financial performance represented by ROA and Tobin’s Q with β = 0.44 at *p* < 0.01 and β = 0.29 at *p* < 0.01, respectively. In addition, SCE has a positive and significant effect on Tobin’s Q with β = 0.41 at *p* < 0.01. These findings are consistent with H2 which states that there is a positive relationship between ICs and financial performance (ROA and Tobin’s Q). However, HCE has a negative and significant relationship with ROA and Tobin’s Q at β = −0.30 at *p* < 0.05 and β = 0.46 at *p* < 0.01, respectively. These findings are inconsistent with H2. Model 3 presents the relationship between firms’ financial performance (ROA and Tobin’s Q) and ENV. Table 5 shows that Tobin’s Q has a negative and significant effect on GOV with β = −0.25 at *p* < 0.01. In addition, ROA has an insignificant impact on SOC. These outcomes do not support H3d which states that the higher the financial performance, the higher the GOV will be. 

Model 4 presents the results of the mediating effect of corporate financial performance on the relationship between ICs and GOV. Model 4 of Table 5 shows that CEE has positive and significant effect on GOV with β = 0.13 at *p* < 0.05. HCE and SCE have an insignificant effect on GOV. ROA shows a negative and significant effect on GOV with β = −0.13 at *p* < 0.05. Moreover, Model 4 of Table 5 shows that CEE has a positive and significant effect on GOV with β = 0.16 at *p* < 0.01. HCE and SCE have an insignificant effect on GOV. Tobin’s Q shows a negative and significant effect on GOV with β = −0.31 at *p* < 0.01. Hence, ROA and Tobin’s Q partially mediate the relationship between ICs and SOC, consistent with the hypothesis of the study. These findings provide evidence that financial performance in terms of the accounting measure and the combination of accounting and market-based measures have an arbitrary impact on the relationship between the ICs and SOC. However, HCE and SCE have an insignificant effect on GOV. Hence, financial performance has no mediating effect on the relationship between these ICs and GOV. The hypotheses results are presented in Table 6.

## 5. Conclusions 

This study investigates the mediating effect of corporate financial performance on the relationship between ICs and CSR of firms the from food industry in Asia. We dwell on prior literature stating that IC and CSR are responsible in value formation for better financial performance. Hence, we proposed that a major issue in the pervasiveness of IC is its impact on CSR and the relationship is mediated by corporate financial performance. We conjecture that the cognizance of the phenomenon is relevant and useful in promoting IC and CSR activities among business firms from the food industry.

This article reveals that intellectual capital and CSR are strategies implemented by firms from the food industry in Asia since improvements in economic value are observed based on composite ratings. Our main findings show that a firm’s investment on capital-employed efficiency generates better composite CSR, ENV, and SOC ratings, which support proponents of the stakeholder theory and resource-based perspective. However, the investments of firms from the food industry in human capital generates lower SOC ratings. In addition, the structural capital efficiency has no significant implication with CSR and its dimensions. Our findings also reveal that a higher CEE generates a better financial performance, both ROA and Tobin’s Q. In addition, a higher SCE generates better financial outcome in terms of Tobin’s Q. However, a higher HCE reflects a lower financial performance both in ROA and Tobin’s Q. Human capital is an employee-related investment to enhance their capabilities and expertise while structural capital is focused on building infrastructure needed by human capital to create value. We conjecture that CEE and SCE are investments which are in line in making more profits for the company. On the other hand, HCE has an immediate effect as it increases costs which lessen the profitability and performance of the company. We infer that this effect can be seen in the long-run as IC components are interrelated in the creation of value for the company.

In terms of financial performance, Tobin’s Q reveals a consistently negative effect on CSR and its pillars while ROA has a favorable effect on SOC. Tobin’s Q is a combination of accounting and market-based estimates of financial performance. These negative effects are associated with the treatment of firms from the food industry as an additional cost due to different compliance and demands from stakeholders. We conjecture that in the long-run, firms from this industry will benefit from CSR initiatives. Lastly, this study asserts that financial performance thru Tobin’s Q partially mediates the relationship between CEE and CSR and its pillars. Moreover, it partially mediates the relationship between other ICs such as HCE, SCE, and SOC. ROA has no mediating effect on ICs and CSR and its pillars. However, it partially mediates the relationship between CEE and GOV. 

This study makes a number of theoretical and practical contributions about the dynamics and evolution of IC, financial performance, and CSR. The present study provides findings which can be used in the cognizance of the phenomenon among corporate citizenship, knowledge-based resources, and value creation for sustainability of doing business in the food industry. Theoretically, we contemplate on an integrated model to explain how intellectual components affect CSR. We include firm financial performance as a mediator to the existing relationship in this important issue at a specific context. Hence, this article provides new empirical evidence from the inconclusive findings from prior literature.

This study also sheds greater light on the importance of IC and socially responsible activities to boost value formation in the food industry context. This industry has substantial visibility to the public and plays a large role in daily human life and the economy and contends with many CSR issues such as food safety, obesity, abuse of alcohol, and packaging management. We suggest that firms from the food industry reflect on these findings as ICs have varied effects on CSR and its pillars. Intangible resources and socially responsible activities should be reported properly to serve as a basis for proper evaluation of the company. Policy makers may reflect on the findings of this research based on the increasing efforts in encouraging CSR engagements in the food industry. A firm’s decision makers should contemplate the idea that CSR initiatives and IC improvements are investments which create a positive image and generate earnings in the long-run. We conjecture that acknowledging the essence of ethical practices with proper management of knowledge resources will lead to greater consumer demand and employee productivity, firm efficiency, and better corporate financial performance. We believe that the cognizance of the phenomenon regarding the investment of firms from the food industry in intellectual capital, compliance and recognition of CSR activities, and profitability and performance, are important issue to ensure food security.

This study examines a limited number of data of food industry firms from the countries available in Thomson Reuters ESG database. We propose that a greater number of representative firms from the food industry in most, if not all, countries in Asia be included for better cognizance of the phenomenon. Our results are subject to verification since the effect of IC and CSR and the mediating effect of financial performance is still inconclusive. Hence, we propose future studies to investigate the magnitude to which this result can be further generalized. Lastly, we suggest that new research examines the impact of the phenomenon in other contexts such as industry and country specifics and creates comparisons for a better understanding of the issue.

## Figures and Tables

**Table 1:** Correlation matrix, means, and standard deviations.

	Mean	SD	*CSR*	*ENV*	*SOC*	*GOV*	*HCE*	*SCE*	*CEE*	*LEV*	*SIZE*	*RDI*	*ROA*	*TOBIN’S*
*CSR*	47.62	11.79	1.00											
*ENV*	53.35	16.49	0.87 ***	1.00										
*SOC*	56.04	14.45	0.93 ***	0.82 ***	1.00									
*GOV*	33.40	12.47	0.61 ***	0.20 ***	0.40 ***	1.00								
*HCE*	11.23	39.56	−0.04	−0.08	−0.05	0.06	1.00							
*SCE*	7.12 × 10^−7^	0.00	−0.09	−0.17 ***	−0.13 **	0.11 **	0.93 ***	1.00						
*CEE*	0.48	0.21	0.15 ***	0.19 ***	0.17 ***	−0.02	0.25 ***	0.16 ***	1.00					
*LEV*	64.36	88.45	0.03	−0.07	−0.01	0.19	−0.10	−0.07	−0.50	1.00				
*SIZE*	19.39	2.09	0.06	0.29 ***	0.19 ***	−0.44 ***	−0.14 **	−0.35 ***	−0.12 **	0.17 ***	1.00			
*RDI*	0.02	0.02	0.12 **	0.02	0.11 **	0.17 ***	−0.11	−0.07	−0.06	0.07	−0.10	1.00		
*ROA*	6.35	5.04	0.14 **	0.05	0.13 **	0.18 ***	0.01	0.03	0.47 ***	−0.26 ***	−0.36 ***	0.09	1.00	
*TOBIN’S*	1.52 × 10^−3^	1.78 × 10^−3^	0.11 **	0.09	0.10	0.09	−0.01	0.03	0.47 ***	−0.35 ***	−0.32 ***	0.15 **	0.75 ***	1.00

Note: ** indicates significance, two-tailed, at the 5% level; *** indicates significance, two-tailed, at the 1% level. LEV: ratio of total liabilities to total assets; SIZE: the natural logarithm of the total assets; RDI: the research and development (R&D) intensity based on the ratio of R&D expenditure to total assets.

**Table 2:** Mediating effect of financial performance (IC and CSR).

	Model 1	Model 2		Model 3		Model 4	
	*(CSR)*	*(ROA)*	*(Tobin’s Q)*	*(CSR)*	*(CSR)*	*(CSR)*	*(CSR)*
*HCE*	−0.20	−0.30	−0.46			−0.22	−0.34
	(−0.94)	(−2.05) **	(−3.17) ***			(−1.01)	(−1.57)
*SCE*	0.18	0.19	0.41			0.19	0.30
	(0.80)	(1.25)	(2.77) ***			(0.84)	(1.35)
*CEE*	0.19	0.44	0.29			0.21	0.28
	(2.67) ***	(8.85) ***	(6.01) ***			(2.65) ***	(3.69) ***
*ROA*				0.05		−0.05	
				(0.68)		(−0.62)	
*TOBIN’S*					−0.18		−0.29
					(−2.22) **		(−3.37) ***
*LEV*	0.18	−0.17	−0.18	0.14	0.08	0.17	0.13
	(2.76) ***	(−3.67) ***	(−3.99) ***	(2.16) **	(1.24)	(2.56) **	(1.96) *
*SIZE*	0.17	0.00	−0.05	0.08	0.03	0.17	0.15
	(1.73) *	(0.01)	(−0.80)	(1.08)	(0.41)	(1.73) *	(1.60)
*RDI*	−0.01	0.09	0.09	−0.02	0.00	0.00	0.02
	(−0.14)	(2.25) **	(2.24) **	(−0.28)	(0.05)	(−0.05)	(0.31)
<Fixed effects>							
Country	Yes	Yes	Yes	Yes	Yes	Yes	Yes
Year	Yes	Yes	Yes	Yes	Yes	Yes	Yes
Adjusted R²	0.22	0.63	0.64	0.21	0.22	0.22	0.25

Note: values per column are the standardized coefficients while *t*-stat values are in parenthesis. * indicates significance, two-tailed, at the 10% level; ** indicates significance, two-tailed, at the 5% level; *** indicates significance, two-tailed, at the 1% level.

**Table 3:** Mediating effect of financial performance (IC and ENV).

	Model 1	Model 2		Model 3		Model 4	
	*(ENV)*	*(ROA)*	*(Tobin’s Q)*	*(ENV)*	*(ENV)*	*(ENV)*	*(ENV)*
*HCE*	−0.28	−0.30	−0.46			−0.29	−0.38
	(−1.35)	(−2.05) **	(−3.17) ***			(−1.39)	(−1.83)
*SCE*	0.27	0.19	0.41			0.28	0.37
	(1.30)	(1.25)	(2.77) ***			(1.32)	(1.72)
*CEE*	0.13	0.44	0.29			0.15	0.20
	(1.95) *	(8.85) ***	(6.01) ***			(1.91) *	(2.74) ***
*ROA*				0.03		−0.03	
				(0.48)		(−0.40)	
*TOBIN’S*					−0.14		−0.22
					(−1.78) *		(−2.65) ***
*LEV*	0.03	−0.17	−0.18	0.01	−0.03	0.02	−0.01
	(0.47)	(−3.67) ***	(−3.99) ***	(0.18)	(−0.57)	(0.37)	(−0.15)
*SIZE*	0.31	0.00	−0.05	0.21	0.18	0.31	0.30
	(3.35) ***	(0.01)	(−0.80)	(2.97) ***	(2.42) **	(3.35) ***	(3.26) ***
*RDI*	−0.07	0.09	0.09	(−0.07	−0.06	−0.07	−0.05
	(−1.19)	(2.25) **	(2.24) **	(−1.22)	(−0.96)	(−1.13)	(−0.85)
<Fixed effects>							
Country	Yes	Yes	Yes	Yes	Yes	Yes	Yes
Year	Yes	Yes	Yes	Yes	Yes	Yes	Yes
Adjusted R²	0.28	0.63	0.64	0.28	0.28	0.28	0.30

Note: values per column are the standardized coefficients while *t*-stat values are in parenthesis. * indicates significance, two-tailed, at the 10% level ** indicates significance, two-tailed, at the 5% level; *** indicates significance, two-tailed, at the 1% level.

**Table 4:** Mediating effect of financial performance (IC and SOC).

	Model 1	Model 2		Model 3		Model 4	
	*(SOC)*	*(ROA)*	*(Tobin’s Q)*	*(SOC)*	*(SOC)*	*(SOC)*	*(SOC)*
*HCE*	−0.36	−0.30	−0.46			−0.35	−0.45
	(−1.74) **	(−2.05) **	(−3.17) ***			(−1.68) *	(−2.15) **
*SCE*	0.33	0.19	0.41			0.33	0.41
	(1.57)	(1.25)	(2.77) ***			(1.53)	(1.94) **
*CEE*	0.25	0.44	0.29			0.24	0.31
	(3.65) ***	(8.85) ***	(6.01) ***			(3.06) ***	(4.26) ***
*ROA*				0.14		0.03	
				(1.96) *		(0.37)	
*TOBIN’S*					−0.07		−0.20
					(−0.92)		(−2.35) **
*LEV*	0.13	−0.17	−0.18	0.11	0.05	0.14	0.10
	(2.09) **	(−3.67) ***	(−3.99) ***	(1.73) *	(0.74)	(2.12) **	(1.51)
*SIZE*	0.39	0.00	−0.05	0.26	0.22	0.39	0.38
	(4.19) ***	(0.01)	(−0.80)	(3.63) ***	(3.01) ***	(4.18) ***	(4.10) ***
*RDI*	0.01	0.09	0.09	0.00	0.02	0.01	0.03
	(0.26)	(2.25) **	(2.24) **	(0.00)	(0.31)	(0.21)	(0.57)
<Fixed effects>							
Country	Yes	Yes	Yes	Yes	Yes	Yes	Yes
Year	Yes	Yes	Yes	Yes	Yes	Yes	Yes
Adjusted R²	0.28	0.63	0.64	0.26	0.25	0.27	0.29

Note: values per column are the standardized coefficients while *t*-stat values are in parenthesis. * indicates significance, two-tailed, at the 10% level ** indicates significance, two-tailed, at the 5% level; *** indicates significance, two-tailed, at the 1% level.

**Table 5:** Mediating effect of financial performance (IC and GOV).

	Model 1	Model 2		Model 3		Model 4	
	*(GOV)*	*(ROA)*	*(Tobin’s Q)*	*(GOV)*	*(GOV)*	*(GOV)*	*(GOV)*
*HCE*	0.21	−0.30	−0.46			0.17	0.07
	(1.21)	(−2.05) **	(−3.17) ***			(0.97)	(0.41)
*SCE*	−0.25	0.19	0.41			−0.22	−0.12
	(−1.40)	(1.25)	(2.77) ***			(−1.26)	(−0.70)
*CEE*	0.07	0.44	0.29			0.13	0.16
	(1.25)	(8.85) ***	(6.01) ***			(2.02) **	(2.71) ***
*ROA*				−0.06		−0.13	
				(−0.98)		(−1.96) **	
*TOBIN’S*					−0.25		−0.31
					(−3.82) ***		(−4.47) ***
*LEV*	0.32	−0.17	−0.18	0.26	0.21	0.30	0.27
	(6.06) ***	(−3.67) ***	(−3.99) ***	(4.96) ***	(4.27) ***	(5.54) ***	(5.06) ***
*SIZE*	−0.40	0.00	−0.05	−0.37	−0.41	−0.40	−0.42
	(−5.15) ***	(0.01)	(−0.80)	(−6.10) ***	(−6.91) ***	(−5.17) ***	(−5.52) ***
*RDI*	0.05	0.09	0.09	0.04	0.06	0.06	0.07
	(0.98)	(2.25) **	(2.24) **	(0.87)	(1.25)	(1.24)	(1.59)
<Fixed effects>							
Country	Yes	Yes	Yes	Yes	Yes	Yes	Yes
Year	Yes	Yes	Yes	Yes	Yes	Yes	Yes
Adjusted R²	0.49	0.63	0.64	0.49	0.51	0.50	0.52

Note: values per column are the standardized coefficients while *t*-stat values are in parenthesis. ** indicates significance, two-tailed, at the 5% level; *** indicates significance, two-tailed, at the 1% level.

**Table 6:** Hypotheses results.

Hypothesis	Results
H1a: There is a positive relationship between ICs and CSR.	
HCE and CSR, SCE and CSR	Reject
CEE and CSR	Accept
H1b: There is a positive relationship between ICs and ENV.	
HCE and ENV, SCE and ENV	Reject
CEE and ENV	Accept
H1c: There is a positive relationship between ICs and SOC.	
HCE and SOC, SCE and SOC	Reject
CEE and SOC	Accept
H1d: There is a positive relationship between ICs and GOV.	
HCE and GOV, SCE and GOV, CEE and GOV	Reject
H2: There is a positive relationship between ICs and financial performance.	
HCE and ROA, SCE and ROA	Reject
CEE and ROA	Accept
HCE and Tobin’s Q, SCE and Tobin’s Q	Reject
CEE and Tobin’s Q	Accept
H3a: The higher the financial performance, the higher will be the CSR ratings.	Reject
H3b: The higher the financial performance, the higher will be the ENV ratings.	Reject
H3c: The higher the financial performance, the higher will be the SOC ratings.	
ROA and SOC	Accept
Tobin’s Q and SOC	Reject
H3d: The higher the financial performance, the higher will be the GOV ratings.	Reject
H4a: Financial performance mediates the relationship between ICs and CSR.	
ROA mediation, HCE and CSR, SCE and CSR, CEE and CSR	Reject
Tobin’s Q mediation, HCE and CSR, SCE and CSR	Reject
CEE and CSR	Partially
H4b: Financial performance mediates the relationship between ICs and ENV.	
ROA mediation, HCE and ENV, SCE and ENV, CEE and ENV	Reject
Tobin’s Q mediation, HCE and ENV, SCE and ENV	Reject
CEE and ENV	Partially
H4c: Financial performance mediates the relationship between ICs and SOC.	
ROA mediation, HCE and SOC, SCE and SOC, CEE and SOC	Reject
Tobin’s Q mediation, HCE and SOC, SCE and SOC, CEE and SOC	Partially
H4d: Financial performance mediates the relationship between ICs and GOV.	
ROA mediation, HCE and GOV, SCE and GOV	Reject
CEE and GOV	Partially
Tobin’s Q mediation, HCE and GOV, SCE and GOV	Reject
CEE and GOV	Partially
